# Supplementary material for: A scaling investigation of urban form features in Latin America cities
Source: PLoS One. 2023 Dec 18;18(12):e0293518. doi: 10.1371/journal.pone.0293518 (PMC10727436; doi:10.1371/journal.pone.0293518)
Supplement: Attachment — Submitted filename: PLOS ONE Reviewer Feedback.pdf [file pone.0293518.s001.pdf]

The paper entitled 'A Scaling Investigation of Urban Form Features of Latin America Cities' examine the investigates the urban scaling behavior in Latin American cities. Particularly, it focuses on the relationship between the physical urban form features with the population size of the cities. In general, the paper contributes interesting findings from the Latin America region. The current manuscript has a few weaknesses.

(1) At the first paragraph of page 3 mentioned about L3 level of analysis. But it does not include in the current study? Meanwhile, 5th Row of the second paragraph of the Descriptive Statistics section – '*Table 3 shows the obtained results using the three L1 city definitions'* is not consistent with the label appears in Table 3 (L1AD, L2, L1UX). Further, the last paragraph of Descriptive Statistics section, L1AM is mentioned without any proper definition. Therefore, I think it would be great to adopt a set of clear and consistent labels for the different level of urban scaling analysis. Perhaps, using an additional figure or table could illustrate the definition ease readers to read and comprehend.

(2) For Scaling Analysis section, the discussion of the result does not truly reflect what it appears from the Table 4 and 5. For an example, the following discussion does not exactly explain the Table 4 results.

'*We note, however, that the Administrative area score with L2 and L1AD definitions, as well as that for Total urban patches with L1AD were quite low (8%, 19%, and 16% respectively).*'
