# Supplementary material for: A scaling investigation of urban form features in Latin America cities
Source: PLoS One. 2023 Dec 18;18(12):e0293518. doi: 10.1371/journal.pone.0293518 (PMC10727436; doi:10.1371/journal.pone.0293518)
Supplement: Attachment — Submitted filename: PLOS ONE Reviewer Feedback_TBT.pdf [file pone.0293518.s003.pdf]

In this version of the paper, I can see some improvements from the previous one. I would like to further highlights my comments as follow:

(1) At the Method section, the terminology of L1 and L2 is mentioned in the discussion. However, there is no proper introduction and definition about them in the first place.

(2) Table 3 contains L1AD density, L2 density and L1UX density. Is it necessary to display them? What is the purpose for having them? I do not find any discussion about these three items.

(3) For Scaling Analysis section, the discussion of the result does not truly reflect what it appears from the Table 4 and 5.

Do make sure the items (examples) below in Scaling Analysis section are consistent with information displayed in Table 4 and 5.

- *'The same occurs for for Number of urban patches in L1AD, with 15.8%'*
- *'The Large road variables has an exponent* $\boldsymbol{\beta}$ *smaller than but very close to 1 in the L2 and L1UX levels.'*

(4) The current Discussion section mainly focuses on the technical discussion – power scaling result, statistical result, and description result, as well as the result relation to other studies. Nonetheless, little discussion on the idea of urban planning is to explain the scaling behavior. Further, the current discussion misses to establish an important connection between the research findings and its implication for better urban planning in Latin America cities.
