# Supplementary material for: A scaling investigation of urban form features in Latin America cities
Source: PLoS One. 2023 Dec 18;18(12):e0293518. doi: 10.1371/journal.pone.0293518 (PMC10727436; doi:10.1371/journal.pone.0293518)
Supplement: Attachment — Submitted filename: Response to Reviewers.docx [file pone.0293518.s002.docx]

1. Please ensure that your manuscript meets PLOS ONE's style requirements, including those for file naming. The PLOS ONE style templates can be found at.

**Response: The manuscript format was placed in the style required by the journal.**

2. Please note that PLOS ONE has specific guidelines on code sharing for submissions in which author-generated code underpins the findings in the manuscript. In these cases, all author-generated code must be made available without restrictions upon publication of the work. Please review our guidelines at <https://journals.plos.org/plosone/s/materials-and-software-sharing#loc-sharing-code> and ensure that your code is shared in a way that follows best practice and facilitates reproducibility and reuse.

**Response: We added a Data availability topic. The scripts are now in a public repository on Github:** <https://github.com/sanchobuendia/Scaling-urban-form>

3. Thank you for stating the following financial disclosure:

The Salud Urbana en América Latina (SALURBAL)/Urban Health in Latin America project was funded by the Wellcome Trust. CIDACS has support from the Wellcome Trust UK (202912/B/16/Z)Moniz, Fundação Oswaldo Cruz, Salvador, Bahia, Brazil.

Please state what role the funders took in the study. If the funders had no role, please state: "The funders had no role in study design, data collection and analysis, decision to publish, or preparation of the manuscript."

If this statement is not correct you must amend it as needed.

Please include this amended Role of Funder statement in your cover letter; we will change the online submission form on your behalf.

**Response: We have fixed this. This reads now:
*Funding***

***The Salud Urbana en América Latina (SALURBAL)/Urban Health in Latin America project was funded by the Wellcome Trust [205177/Z/16/Z]. CIDACS has support from the Wellcome Trust UK (202912/B/16/Z) Instituto Gonçalo Moniz, Fundação Oswaldo Cruz, Salvador, Bahia, Brazil. UB was also supported by Office of the Director of the National Institutes of Health under award number DP5OD026429. The funders had no role in study design, data collection and analysis, decision to publish, or preparation of the manuscript.***

4. Thank you for stating the following in the Acknowledgments Section of your manuscript:

The Salud Urbana en América Latina (SALURBAL)/Urban Health in Latin America project is funded by the Wellcome Trust [205177/Z/16/Z].

However, funding information should not appear in the Acknowledgments section or other areas of your manuscript. We will only publish funding information present in the Funding Statement section of the online submission form.

Please remove any funding-related text from the manuscript and let us know how you would like to update your Funding Statement. Currently, your Funding Statement reads as follows:

The Salud Urbana en América Latina (SALURBAL)/Urban Health in Latin America project was funded by the Wellcome Trust. CIDACS has support from the Wellcome Trust UK (202912/B/16/Z)Moniz, Fundação Oswaldo Cruz, Salvador, Bahia, Brazil.

Please include your amended statements within your cover letter; we will change the online submission form on your behalf.

**Response: This part has been deleted from the text.**

5. We note that you have indicated that data from this study are available upon request. PLOS only allows data to be available upon request if there are legal or ethical restrictions on sharing data publicly. For more information on unacceptable data access restrictions, please see <http://journals.plos.org/plosone/s/data-availability#loc-unacceptable-data-access-restrictions>.

In your revised cover letter, please address the following prompts:

a) If there are ethical or legal restrictions on sharing a de-identified data set, please explain them in detail (e.g., data contain potentially sensitive information, data are owned by a third-party organization, etc.) and who has imposed them (e.g., an ethics committee). Please also provide contact information for a data access committee, ethics committee, or other institutional body to which data requests may be sent.

**Response: There are not ethical or legal restrictions**

b) If there are no restrictions, please upload the minimal anonymized data set necessary to replicate your study findings as either Supporting Information files or to a stable, public repository and provide us with the relevant URLs, DOIs, or accession numbers. For a list of acceptable repositories, please see <http://journals.plos.org/plosone/s/data-availability#loc-recommended-repositories>.

**Response: Data availability The SALURBAL project welcomes queries from anyone interested in learning more about its dataset and potential access to data. To learn more about SALURBAL’s dataset, visit https://drexel.edu/lac/ or contact the project at** [**salurbal@drexel.edu**](mailto:salurbal@drexel.edu)**. Furthermore, we are creating an open data repository to which we are adding the script, and data will be available at** [**https://data.lacurbanhealth.org**](https://data.lacurbanhealth.org)

6. Your ethics statement should only appear in the Methods section of your manuscript. If your ethics statement is written in any section besides the Methods, please move it to the Methods section and delete it from any other section. Please ensure that your ethics statement is included in your manuscript, as the ethics statement entered into the online submission form will not be published alongside your manuscript.

**Response: we have moved the ethical statement to the methods section.**

7. We note that Figure 1 in your submission contain [map/satellite] images which may be copyrighted. All PLOS content is published under the Creative Commons Attribution License (CC BY 4.0), which means that the manuscript, images, and Supporting Information files will be freely available online, and any third party is permitted to access, download, copy, distribute, and use these materials in any way, even commercially, with proper attribution. For these reasons, we cannot publish previously copyrighted maps or satellite images created using proprietary data, such as Google software (Google Maps, Street View, and Earth). For more information, see our copyright guidelines: <http://journals.plos.org/plosone/s/licenses-and-copyright>.

**Response: The figure was created in free software using non-copyrighted material. Therefore, it does not need copyright. We only include the name of the software in the text.**

# Reviewer 1:

**Response: We thank the reviewer for the comments, which we understand to be directed mainly to the text in the first section Introduction. In the answers to the formulated questions we indicate the specific actions and changes that were made.**

1) The manuscript is a straightforward application of the urban scaling framework --- with insufficient argumentation as to why urban form should exhibit scaling behavior. Vague invocations of cities as complex systems---a claim which is not at all informative---do not constitute an argument as to why urban form should conform to a scaling relationship.

**Response: The first paragraph now presents only a general landscape of the research on city growth phenomena, finalizing with the mention of the scaling analysis framework. The mention to theoretical support for scaling ideas was moved to the next paragraph. We hope this provides a better justification for this study.**

2) There is an established argument (derivation) as to why urban infrastructure should exhibit scaling behavior---see Lobo et al (2020) in Urban Studies. The manuscript exhibits ignorance of recent work in urban scaling.

**Response: Thank you for the suggestions. The second paragraph has been rewritten, including now a substantial discussion on the theoretical support for scaling approaches. The corresponding references are introduced, including the specific item suggested above. We also included a comment on temporal scaling analysis and added three new references, including Lobo et al.**

3) The authors refer to a non existing controversy regarding the importance of urban delineation for investigating scaling: cities as social networks embedded in physical space should exhibit scaling; but not administratively defined city entities.

**Response: Others (e.g., Cottineau, Hatna, Arcaute and Batty, 2017 –see** <https://www.sciencedirect.com/science/article/pii/S0198971516300448> **) do suggest this is a controversy. It seems that there is disagreement on whether this is controversial.**

**The mention to the indicated controversy was removed from paragraph 6. In the paragraph 9, when we describe the different city definitions available on the Salurbal data sets, we indicate we carried out scaling analysis based on cities urban extents as detected by satellite imagery as well as on admistrative definition. We briefly comment the expected results taking into account the reviewer’s remarks above.**

4) The connection of the presented results and urban planning is superficial and does not reflect how scaling results can inform urban policy.

**Response: We seek to bring a more in-depth discussion on this subject in paragraph 4, indicating more clearly how the results of the scale analysis can help policy makers in making decisions to improve the quality of life of all populations and subgroups.**

5) The study of urban scaling over time is a topic of active work which the authors do not invoke; see, for example, <https://royalsocietypublishing.org/doi/pdf/10.1098/rsif.2019.0846>.

**Response: We thank the reviewer for the suggestions. As indicated in our answer to query 2, we added a brief comment on this relevant aspect in the new paragraph 7.**

# Reviewer 2:

The paper entitled ‘A Scaling Investigation of Urban Form Features of Latin America Cities’ examine the investigates the urban scaling behavior in Latin American cities. Particularly, it focuses on the relationship between the physical urban form features with the population size of the cities. In general, the paper contributes interesting findings from the Latin America region. The current manuscript has a few weaknesses.

(1) At the first paragraph of page 3 mentioned about L3 level of analysis. But it does not include in the current study? Meanwhile, 5th Row of the second paragraph of the Descriptive Statistics section – ‘Table 3 shows the obtained results using the three L1 city definitions’ is not consistent with the label appears in Table 3 (L1AD, L2, L1UX). Further, the last paragraph of Descriptive Statistics section, L1AM is mentioned without any proper definition. Therefore, I think it would be great to adopt a set of clear and consistent labels for the different level of urban scaling analysis. Perhaps, using an additional figure or table could illustrate the definition ease readers to read and comprehend.

**Response**: **These inconsistencies were identified and we included an explanation about L3, changed the description of Table 3, dropped the L1AM term and improved the description of levels.**

(2) For Scaling Analysis section, the discussion of the result does not truly reflect what it appears from the Table 4 and 5. For an example, the following discussion does not exactly explain the Table 4 results.

‘We note, however, that the Administrative area score with L2 and L1AD definitions, as well as that for Total urban patches with L1AD were quite low (8%, 19%, and 16% respectively).’

**Response**: **We adjusted the interpretation of the results in Tables 4 and 5. First, the scaling parameters of Table 4 was analyzed in two separate paragraphs (second and third paragraphs of the section). Second, the interpretation of Table 5 also was developed in a new paragraph (sixth paragraph of section).**
