# Supplementary material for: A scaling investigation of urban form features in Latin America cities
Source: PLoS One. 2023 Dec 18;18(12):e0293518. doi: 10.1371/journal.pone.0293518 (PMC10727436; doi:10.1371/journal.pone.0293518)
Supplement: Attachment — Submitted filename: Response to reviews.docx [file pone.0293518.s004.docx]

Dear reviewer, we thank you for all your comments and suggestions aimed at improving our article. Below are the answers.

6. Review Comments to the Author

Please use the space provided to explain your answers to the questions above. You may also include additional comments for the author, including concerns about dual publication, research ethics, or publication ethics. (Please upload your review as an attachment if it exceeds 20,000 characters)

Reviewer #2: In this version of the paper, I can see some improvements from the previous one. I would like to further highlights my comments as follow:

(1) At the Method section, the terminology of L1 and L2 is mentioned in the discussion. However, there is no proper introduction and definition about them in the first place.
Response: Thanks for this remark. We removed any reference about L1 and L2 in the Method section. However, we note that the definition of “L1” and “L2” is available in the Data section together with the reference to the seminal project protocol article, as follows: “Being more specific, we restrain our study to urban agglomerations classified as “cities” at the L1 and L2 SALURBAL levels [36], which amounts to taking *n=*371 or 1436 at the L1 or L2 levels, respectively”. (Quistberg et al. Building a data platform for cross-country urban health studies: the SALURBAL study. Journal of urban health. 2019 Apr 15;96:311-37).

(2) Table 3 contains L1AD density, L2 density and L1UX density. Is it necessary to display them? What is the purpose for having them? I do not find any discussion about these three items.

Response: Thanks for this comment. We reformatted Table 3 to present only the results at the L1AD, L2 and L1UX level. The discussion of the items encompasses the paragraphs requested by the reviewer’s comment (4) on questions about urban planning. Please see specific answer to comment 4 below.

(3) For Scaling Analysis section, the discussion of the result does not truly reflect what it appears from the Table 4 and 5.

Do make sure the items (examples) below in Scaling Analysis section are consistent with information displayed in Table 4 and 5.

- ‘The same occurs for Number of urban patches in L1AD, with 15.8%’

Thanks for highlighting this. Fixed for L2. “The same occurs for Number of urban patches in L2, with 15.8%.”

- ‘The Large Road variables has an exponent smaller than but very close to 1 in the L2 and L1UX levels.’
Thanks for that. It is fixed: “The large road variable has an exponent β very close to 1 in the L1AD and L1UX levels”.

(4) The current Discussion section mainly focuses on the technical discussion – power scaling result, statistical result, and description result, as well as the result relation to other studies. Nonetheless, little discussion on the idea of urban planning is to explain the scaling behavior. Further, the current discussion misses to establish an important connection between the research findings and its implication for better urban planning in Latin America cities.

Response: Thanks for this comment. As mentioned, we inserted the following sentences in the discussion section:

“The results found here will serve as a basis for comparison with further studies targeted to similar analyses for cities in other regions. We emphasize the importance of using appropriate city definitions in scaling analyses. Our study reveals that city definitions based on the actual extent of the urban area yield more accurate insights compared to administrative definitions. This highlights the need for urban planners in Latin America to consider the physical boundaries of urban areas when making decisions about resource allocation, spatial organization, and infrastructure development. By understanding the scaling behavior of built environment variables in relation to the actual urban extent, planners can create more effective strategies to address the specific needs and challenges of rapidly expanding cities.

Furthermore, the study's discovery of a degree of universality in the relationship between population size and built environment variables across diverse Latin American cities has important implications for urban planning. Despite varying economic, historical, and cultural differences, the fundamental mechanisms that drive urban growth appear to exhibit common patterns. This finding suggests that certain strategies for city development may be applicable across different cities in the region, providing valuable guidance for planners seeking sustainable and efficient urbanization approaches.”
